# Advances in deubiquitinating enzymes in lung adenocarcinoma

**DOI:** 10.7150/jca.56532

**Published:** 2021-07-25

**Authors:** Xi-Jia Zhou, Rui Li, Xiao Liu, Yi-Qing Qu

**Affiliations:** 1 Department of Pulmonary and Critical Care Medicine, Qilu Hospital, Cheeloo College of Medicine, Shandong University (Jinan 250012, China); 2 Department of Pulmonary and Critical Care Medicine, Qilu Hospital of Shandong University (Jinan 250012, China)

**Keywords:** deubiquitinating enzymes, lung adenocarcinoma, deubiquitinase inhibitor, targeted therapy

## Abstract

The process of ubiquitination and deubiquitination is widely present in the human body's protein reactions and plays versatile roles in multiple diseases. Deubiquitinating enzymes (DUBs) are significant regulators of this process, which cleave the ubiquitin (Ub) moiety from various substrates and maintain protein stability. Lung adenocarcinoma (LUAD) is the most common type of non-small cell lung cancer (NSCLC) and remains refractory to treatment. To elucidate the mechanism of LUAD and advance new therapeutic targets, we review the latest research progress on DUBs in LUAD. We summarize the biological capabilities of these DUBs and further highlight those DUBs that may serve as anticancer target candidates for precision treatment. We also discuss deubiquitinase inhibitors, which are expected to play a role in targeted LUAD therapy.

## Introduction

Lung cancer is a disease with the highest incidence and mortality worldwide. Lung adenocarcinoma (LUAD) is the most common subtype of lung cancer, which has characteristics of complex mechanisms, aggressiveness, and poor prognosis^1^-^3^. Although surgical treatment, radiotherapy, chemotherapy, and targeted, comprehensive therapy have made significant progress over the past decade, the five-year survival rate remains bleak^4^. Therefore, it is compelling to investigate the mechanisms of LUAD in order to identify new therapeutic targets to improve future treatment decisions^5^, ^6^.

Among existing achievements, a breakthrough in tumor research has been the discovery of many proto-oncogenes and tumor suppressor genes^7^, ^8^. Mutation, fusion, deletion, overexpression, and suppression of these genes affect the biological process of tumor cells^9^-^11^. However, studies have proven that expression level of mRNA do not truly reflect protein expression^12^. Post-translational modifications (PTMs) of proteins refer the covalent modification of individual amino acid residues on a protein after the translation process is complete. PTMs further promote an increase in complexity from the genomic level to the proteome. Primary forms of PTMs include phosphorylation, glycosylation, acetylation, and ubiquitination^13^, ^14^.

Ubiquitination is the covalent binding of ubiquitin (Ub) to a target protein under the catalysis of a series of enzymes^15^, ^16^. To be precise, ubiquitination is a kind of intracellular protein labelling system, which attach different labels to target proteins for the purpose of explicitly degrading proteins^17^. The action of three types of enzymes achieves this protein degradation mechanism: ubiquitin-activating enzymes (E1), ubiquitin-conjugating enzymes (E2), and ubiquitin ligases (E3). Among them, E3 is the most abundant enzyme type and is primarily responsible for ubiquitination specificity. There are more than 600 types of E3 ligases in the human body, which recognize substrate proteins and combine with E2-Ub conjugates^18^. Deubiquitinating enzymes (DUBs) cleave the covalent bonds between Ub and substrate proteins. During the process of PTM, deubiquitination and ubiquitination reactions usually maintain a dynamic balance^19^.

**Figure 1:**
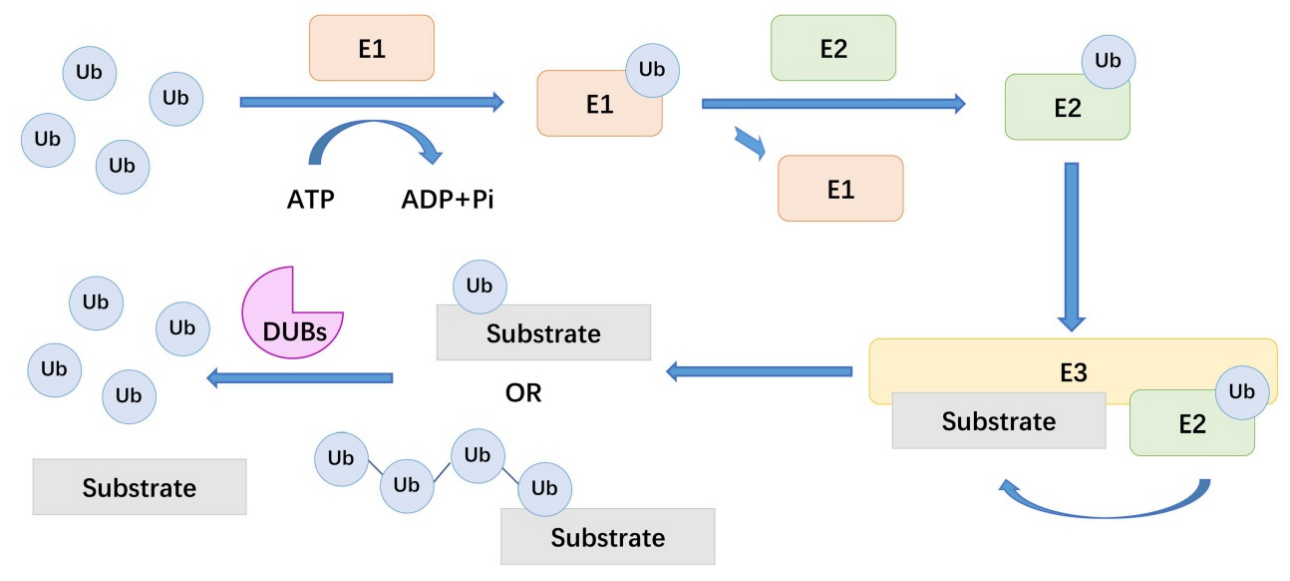
The general mechanism of DUBs. Ub: ubiquitin, E1: ubiquitin-activating enzymes, E2: ubiquitin-conjugating enzymes, E3: ubiquitin ligases, DUBs: deubiquitinating enzymes.

There are about 100 types of DUBs in the human body. These enzymes can be divided into seven families based on the active site of DUBs, including ubiquitin-specific proteases (USPs), ubiquitin COOH-terminal hydrolases (UCHs), ovarian tumor proteases (OTUs), Josephine, the JAB1 / MPN / MOV34 family (JAMMs) and motif interacting with Ub-containing novel DUB family (MINDYs)^20^, ^21^. DUBs are enzymes that act between ubiquitin molecules or between ubiquitin molecules and substrate proteins^22^. In the process of ubiquitination, Ub provides eight attachment sites for the formation of polymeric chains, including seven lysine residues, K6, K11, K27, K29, K33, K48, K63 and its amino terminus. For example, K48-linked chain is considered to be related to the proteasomal degradation of the substrate. And DUBs usually deubiquitinate substrate proteins by removing these ubiquitin chains^23^. Here, we summarize a mechanistic diagram of ubiquitination and deubiquitination (Figure 1). By reversing the ubiquitination process, DUBs modulate a variety of cellular processes, including protein degradation, DNA damage and repair, endocytosis, apoptosis, cell cycle, and receptor signal transduction^24^-^27^. Therefore, the regulatory effect of DUBs in LUAD, especially the potential therapeutic value of DUBs on LUAD treatment, has attracted increasing attention^21^, ^28^.

## A brief account of DUBs

As significant regulators, DUBs are involved in various diseases, including neurodegenerative disorders, inflammation, and cancer^23^, ^27^, ^29^, ^30^. For example, UCHL1 plays a significant role in promoting corticospinal motor neuron (CSMN) stability by maintaining protein homeostasis^31^. USP7 and USP47 regulate the ubiquitination state of the NLR family pyrin domain containing 3 (NLRP3) and influence inflammasome activation in macrophages^32^.

**Figure 2:**
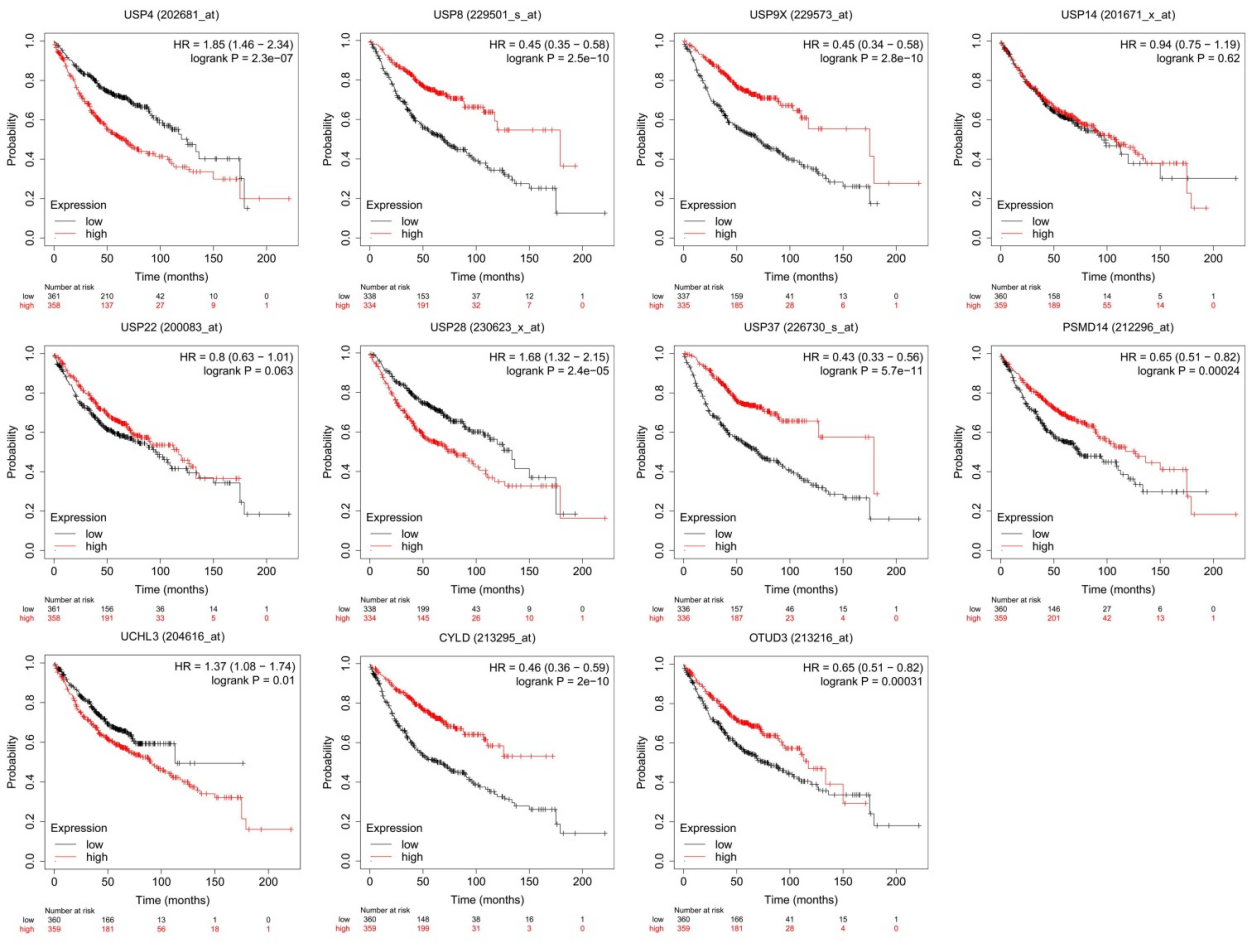
Prognostic value of DUBs. We used Kaplan-Meier Plotter to analyze the prognostic value of DUBs. We searched the gene symbols, divided the LUAD patients into high-expression and low-expression groups based on the median and analyzed the difference in overall survival (OS) between the two groups. Some DUBs, for example, USP4, USP8, USP9X, are valuable in predicting the prognosis of LUAD patients.

However, the most relevant research on DUBs is in the field of cancer^33^, ^34^, including lung, liver^35^, breast^36^, stomach^37^, colon^38^, prostate^39^ and ovarian cancers^40^. In this area, the mechanisms of DUBs in different tumors are described, and their potential as cancer therapeutics has attracted attention. For example, USP21-mediated deubiquitination of mitogen-activated protein kinase 2 (MEK2) promotes liver cancer cell proliferation in vitro and tumor growth in vivo^41^. BAP1 interacts with Krüppel-like factor 5 (KLF5) to stabilize KLF5 and promotes proliferation of breast cancer cells^42^. USP42 promotes the proliferation and invasion of gastric cancer cells by affecting matrix metalloproteinases (MMPs) and epithelial-mesenchymal transition (EMT)^43^. Further research focuses on the therapeutic effect of DUBs and their inhibitors on tumor therapy. For example, USP37 is overexpressed in breast cancer and promotes cell migration, invasion and EMT by regulating hedgehog signaling pathway. Downregulating of USP37 increases the sensitivity of breast cancer cells to cisplatin^44^. HBX19818 and P22077, two inhibitors that target USP10, induce anti-proliferative effects against FMS-like tyrosine kinase 3 (FLT3) mutant acute myeloid leukemia (AML)^45^. In this review, we concentrate on the particular role of DUBs in LUAD (Table 1 and Table 2). And the prognostic value of some essential DUBs was analyzed using Kaplan-Meier Plotter (http://kmplot.com/analysis/) (Figure 2).

## DUBs affect proliferation and apoptosis in LUAD

The most fundamental feature of cancer cells is their infinite proliferation ability. Cancer cells control the release of growth-promoting signals and autonomously regulate their own growth^46^, ^47^. DUBs affect signaling pathways and regulatory factors related to LUAD cell proliferation and apoptosis.

### USP8

USP8 is an essential cell growth-related DUB. USP8 maintains embryonic stem cell (ESC) stemness by deubiquitinating ectopic P-granules autophagy protein five homologs (EPG5). EPG5 is a specific autophagy protein, which protects pluripotency in ESCs by binding to LC3. USP8 directly deubiquitinates EPG5 by removing K63-linked ubiquitin and enhance the interaction between LC3 and EPG5^48^.

In LUAD, USP8 stabilizes receptor tyrosine kinases (RTKs), contributes to proliferative activity, and inhibits apoptosis in A549 cells^49^, ^50^. Hyperactivation of AKT in LUAD promotes the combination of USP8 and stratifin (SFN). SFN-USP8 complex deubiquitinates RTKs and facilitates recycling of RTKs to the plasma membrane. The increase of RTKs leads to excessive activation of some signaling pathways, such as epidermal growth factor receptor (EGFR) signaling, and causes cell proliferation. Compared with normal lung tissue, USP8 is highly expressed in LUAD tissue, and is related to pathological subtype, lymphatic permeation, vascular invasion and EGFR overexpression of tumor tissue. Patients with high expression of USP8 have a significantly poorer prognosis, suggesting that USP8 has the potential as a prognostic marker. Furthermore, it has been reported that USP8 may represent a potential therapeutic target for gefitinib-resistant LUAD. Inhibition of USP8 activity significantly reduces the activity of gefitinib-resistant lung adenocarcinoma cell lines^51^.

### USP14

USP14 is a DUB associated with viral infection, neurodegenerative diseases and cancers^52^. USP14 has been reported to be overexpressed in LUAD and promote proliferation through the accumulation of β-catenin. β-catenin is a vital member of the Wnt-pathway that promotes proliferation in cancer cells. Silencing USP14 causes a sharp drop in β-catenin protein levels. Moreover, experiments confirmed that after knocking down USP14, the number of cells in the S phase significantly decreased (p<0.05), and the number of cells in the G0/G1 phase significantly increased (p<0.05). Meanwhile, expression of USP14 is significantly correlated with the prognosis of LUAD patients^53^. Therefore, USP14 may become a diagnostic marker and therapeutic target for LUAD patients. 1-[1-(4-fluorophenyl)-2,5-dimethylpyrrol-3-yl]-2-pyrrolidin-1-ylethanone, also known as IU1, is a kind of specific small-molecule inhibitor of USP14^54^. IU1 and its analogs can bound to USP14 and prevent the C-terminus of ubiquitin from approaching the catalytic center^55^. The results of in vitro experiments have confirmed that IU1-47 can inhibit the proliferation, invasion and migration of lung cancer cells^56^.

### USP17

USP17 regulates cell cycle progression by deubiquitinating cyclin A. Knockdown of USP17 decreased cyclin A levels. Cyclin A is a critical molecule that regulates the cell cycle^57^. USP17 interacts with cyclin A and removes the Ub chains conjugated onto cyclin A to stabilize it. Through this mechanism, USP17 drives cell cycle from G0/G1 to S phase and mediates the proliferation of LUAD cells^58^. Interestingly, USP17 has also been reported to drive a positive feedback loop between macrophages and LUAD cells. USP17 disrupts TRAF2/TRAF3 complex formation, enhancing inflammation and stemness in LUAD cells and promoting cell growth^59^. It is worth noting that USP17 is significantly highly expressed in NSCLC tissues and cells. In vivo experiments have also confirmed that knocking down USP17 inhibits the tumorigenesis of NSCLC^60^. Therefore, USP17 is a potential target for LUAD targeted therapy, which is worthy of further study.

### USP22

USP22 is a DUB related to cell cycle regulation, tumor progression and EMT^61^. The expression of USP22 is significantly increased in LUAD tissues, suggesting that USP22 is a potential diagnostic marker of LUAD. In EGFR-mutated LUAD, USP22 positively regulates cell proliferation, G1/S phase transformation and metastasis. USP22 deubiquitinating EGFR localized on late endosomes, which stabilizes the EGFR protein, and increases the recovery and utilization of EGFR. Meanwhile, USP22 also promotes phosphorylation of EGFR downstream molecules. More importantly, USP22 promotes the resistance of EGFR-mutant lung ADC cells to EGFR-TKI, which provides new treatment strategies for patients with EGFR mutations^62^.

### PSMD14

Proteasome 26S subunit, non-ATPase 14 (PSMD14) is a DUB associated with the development of multiple tumors. For example, PSMD14 stabilizes ALK2 and contributes to tumor growth of colorectal cancers^63^. It has been reported that PSMD14 is upregulated in LUAD, and its high expression is related to a poor prognosis in LUAD patients. Knocking down PSMD14 inhibits proliferation of LUAD cells and induces cell cycle arrest and apoptosis. In H1299 and A549 cell lines, knocking down PSMD14 can induce G1 phase cell cycle arrest and cell senescence in both cell lines to inhibit cell survival. Among them, in the H1299 cell line, depletion of PSMD14 also leads to apoptosis in some cells^64^. In colorectal cancers, PSMD14 deubiquitinates K48-linked chains on ALK2 type I receptor to stabilize ALK2, resulting in the increases of cancer chemoresistance^63^. This provides a feasible direction for the study of PSMD14 in LUAD.

### BAP1

BRCA1 related protein 1 (BAP1) is an essential nuclear localization DUB that inhibits lung cancer proliferation and promotes apoptosis. Experiments demonstrated that BAP1 is inhibited by the non-coding RNA miR-31. Combined with the prediction results of computer algorithms, such as TargetScan (http://www.targetscan.org/vert_72/), it was speculated that BAP1 represents a potential target of miR-31, and this conjecture was verified through Western blot and luciferase reporter assay^65^. In addition, some studies indicate that BAP1 mutation predisposes to LUAD^66^. Studies have shown that BAP1 inhibits the expression of solute carrier family 7 member 11 (SLC7A11) by decreasing H2Aub occupancy on it. And downregulation of SLC7A11 leads to ferroptosis, a non-apoptotic form of cell death, and inhibit tumor growth^67^. Unfortunately, the specific mechanism of BAP1 in LUAD has not been elucidated.

### OTUD3

With respect to OTUs, it has been reported that OTUD3 accelerates the growth of human lung cancer. In LUAD, OTUD3 interacts with the glucose-regulated protein 78 (GRP78) and deubiquitinates it. GRP78 is a multifunctional protein and promotes tumor growth and metastasis. OTUD3 positively regulates GRP78, which in turn promotes the growth of LUAD cells^68^. Studies have shown that OTUD3 cleaves Lys6 and Lys11-linked diubiquitin^69^, but the molecular mechanism of OTUD3 in LUAD is still unclear. It is worth mentioning that OTUD3 is decreased in breast cancer and suppresses tumorigenesis by deubiquitinating PTEN 70. In contrast, OTUD3 is high-expressed in LUAD and indicates poorer overall survival for patients. Therefore, OTUD3 has the potential as a specific marker for diagnosing LUAD.

## DUBs affect invasion and migration in LUAD

An important reason for the high rate of tumor recurrence and mortality is that tumor cells have a high incidence of invasion and migration. These tumor cells are different from normal cells with respect to cell metabolism, tumor microenvironment, and activation of signaling pathways^71^. In LUAD, some DUBs affect both tumor cell proliferation and migration. For example, USP22 mentioned above promotes LUAD cell invasion and migration^62^. It also promotes angiogenesis, proliferation, EMT, KRAS proto-oncogene (KRAS), and transcriptional regulator Myc-like (c-Myc) pathways in LUAD cells^72^. And OTUD3 also has specific functions to promote metastasis of LUAD cells^68^.

### USP4

USP4 is a member of the USPs family and affects the occurrence, invasion and migration of tumors. Through bioinformatics analysis in The Cancer Genome Atlas (TCGA) database, it was found that in LUAD tissues, expression of USP4 was significantly decreased. At the same time, USP4 expression was reported as a favorable independent prognostic factor for overall survival (OS) and recurrence-free survival (RFS) in LUAD patients^73^. USP4 influences brain metastasis of LUAD through β-catenin stabilization. β-catenin is a key molecule essential for acquisition of metastatic potential. Knockdown of USP4 reduces the level of β-catenin protein without affecting the level of mRNA expression. Therefore, it can be speculated that USP4 promotes brain metastasis of LUAD cells by stabilizing the β-catenin protein^74^. USP4 could be a potential therapeutic target for metastatic LUAD.

### USP9X

USP9X is another DUB reported to be associated with LUAD invasion. Prostaglandin E synthase (PTGES) plays a key role in this function. PTGES, also known as mPGES-1, is an enzyme that promotes the conversion of prostaglandin H2 (PGH2) to prostaglandin E2 (PGE2)^75^. At the same time, PTGES is a key molecule related to the stemness of LUAD. Knocking down PTGES in LUAD cell lines inhibits cell invasion and migration. USP9X-mediated deubiquitinating reaction stabilized PTGES, and increased its expression in LUAD. Therefore, USP9X promotes the metastasis of tumor cells^76^. Another possible mechanism is that USP9X deubiquitinates dual specificity protein kinase TTK on K48-linked Ub chains. USP9X and TKK promote proliferation, invasion and migration in LUAD cells^77^.

Interestingly, USP9X exerts different functions in different cancers. In colorectal cancer, USP9X stabilizes FBW7 and suppresses the cancer^78^. In another highly aggressive tumor, glioblastoma (GBM), USP9X interacts with aldehyde dehydrogenase one family member A3 (ALDH1A3) and stabilizes it to promote the tumorigenic capacity of mesenchymal stem cells^79^. These experiments suggest that USP9X could be a therapeutic target for various cancers. Inhibition of USP9X may play an important role in the treatment of metastatic LUAD.

### USP37

EMT is the process in which epithelial cells acquire interstitial characteristics through specific procedures. EMT is related to tumor occurrence, invasion, migration, and resistance to treatment^80^. Snail protein is an essential positive regulator of EMT, which is upregulated by USP37. USP37 and Snail co-localize in the nucleus. USP37 deubiquitinates Snail protein, prevents the degradation of Snail, and stabilizes the initially unstable Snail protein. In this way, USP37 achieves the function of promoting the invasion and migration of LUAD cells^81^. It is worth mentioning that USP37 also deubiquitinates and stabilizes c-Myc to affect proliferation in cancer cells^82^. USP37 levels is significantly increased in LUAD tissues, which provides a clue that USP37 could be a specific molecule for LUAD diagnosis.

### UCHL3

UCHL3 is a DUB belonging to the UCHs family. UCHL3 deubiquitinates chromatin modifier lymphoid-specific helicase (LSH). LSH is critical for the malignant progression of cancers by promoting cell proliferation and migration. Thus, UCHL3 contributes to the occurrence and transfer of LUAD. At the same time, the lncRNA GIAT4RA counteracts the deubiquitinating effects of LSH by interfering with interaction between LSH and UCH-L3, reducing levels of LSH protein, and playing the role in inhibiting the invasion and migration of LUAD cells^83^. Another article proposed that UCHL3 promotes the growth and stem-like properties of NSCLC cells through deubiquitinating and stabilizing Aryl hydrocarbon receptor (AhR)^84^. UCHL3 is significantly high-expressed in LUAD and associated with poor survival in LUAD. These data all indicate that UCHL3 could be a potential target for the treatment of metastatic LUAD.

## DUBs and LUAD treatment

At present, there are many treatment methods for LUAD, including traditional surgical treatment, radiotherapy, and chemotherapy^85^, ^86^. Moreover, molecular targeted therapy is an essential part of precision treatment for LUAD^87^, ^88^. For example, in patients with EGFR mutations, gefitinib, erlotinib, afatinib, and osimertinib can be chosen clinically^89^. However, targets deficiency, drug resistance, and drug toxicity affect the clinical efficacy of molecular targeted therapies. Some astudies suggest that USP4^90^, USP17^60^, USP18^91^, USP21^92^, OTUD1, OTUD3, and OTUD4^93^ may represent potential targets for targeted therapy of LUAD. DUBs are significant regulators in LUAD and have attracted much attention in the field of targeted drug development.

### USP7

Macrophages (MΦs) mainly differentiate into two phenotypes, M1 MΦs is a tumor suppressor subtype, and M2 MΦs is a tumor promoting subtype. Studies have found that the inhibiting of USP17 plays an important role in reprogramming tumor associated macrophages (TAMs) to M1 MΦs through p38 MAPK pathway. Since this reprogramming of TAMs has the effect of eliminating tumor cells, the treatment targeting USP17 may play an important role in the treatment of lung cancer. P5091, a kind of USP7 inhibitor, can activate the anti-tumor immune responses. At the same time, P5091 and anti-pd-L1 have a synergistic anti-tumor effect in vivo. Therefore, P5091 may play an important role in LUAD immunotherapy^94^.

### USP9X

USP9X inhibition was shown to increase tumor cell sensitivity to various chemotherapeutic agents, especially Bcl-2/Bcl-xL inhibitors. Bcl-xL and Mcl-1, members of the Bcl-2 family, convey resistance to drug therapy in many tumors. USP9X positively promotes the expression of Mcl-1. In contrast, the USP9X inhibitor WP1130 promotes the degradation of Mcl-1. Therefore, the combined use of WP1130 and Bcl-xL inhibitors may play a synergistic role in promoting apoptosis of LUAD cells^95^.

### USP28

In tumor cells, USP28 antagonizes the activity of the F-box and WD repeat domain containing 7 (FBXW7) to promoting the stability of c-Myc. As a target protein recognition component of the E3 complex, FBXW7 is involved in the degradation of various oncogenes, including cyclins and c-Myc. Moreover, USP28 is upregulated in LUAD and related to poor prognosis. Therefore, we suggest that USP28 can be used as a targeted therapy for LUAD^96^, ^97^. [1,2,3]triazolo[4,5-d]pyrimidine derivatives are highly effective and selective USP28 inhibitors and have the potential as targeted therapy drugs for LUAD patients^98^.

### CYLD

Tumor necrosis factor-related apoptosis-inducing ligand (TRAIL) is a member of the tumor necrosis factor (TNF) family. It has become a vital tumor biotherapeutic factor due to its ability to induce apoptosis of malignant cells. However, many tumor cells are resistant to TRAIL-induced apoptosis. Overexpression of CYLD lysine 63 deubiquitinase (CYLD) blocks TRAIL-induced nuclear factor-kappa B (NF-κB) activation, increases TRAIL-induced apoptosis of lung cancer cells and sensitizes lung cancer cells to TRAIL-induced apoptosis. Therefore, it has been suggested that CYLD is a therapeutic factor for lung cancer, especially when treating patients with high NF-κB activity^99^-^101^.

### Deubiquitinase inhibitors

Although they are still in preclinical stages, some deubiquitinase inhibitors have proven to have significant tumor-suppressing effects laboratory experiments^102^-^104^. According to a new report, a new gold(I) complex, Au(PPh3)PT, has been synthesized. Au (PPh3) PT selectively inhibits 19S proteasome-related subunits, such as UCHL5 and USP14, reducing hydrolysis of related proteins, and inducing the apoptosis in two lung cancer cell lines, A549 and H1299. In vivo experiments demonstrated that Au (PPh3) PT could also effectively inhibit the growth of transplanted tumors in LUAD in nude mice^105^. At present, research on deubiquitinase inhibitors is a hot topic in the field of anticancer drugs, and there are many related articles (Table 3). For example, ADT triggers the accumulation of ubiquitinated proteins in cells, effectively inhibiting proteasome subtypes USP14 and UCHL5 and inducing tumor cell apoptosis^106^. AC17 acts as an irreversible deubiquitinase inhibitor of 19S RP, leading to inhibition of the NF-κB pathway and reactivation of the pro-apoptotic protein p53^107^.

## Conclusions

This review focuses on research of DUBs in LUAD. On the one hand, we demonstrate the mechanisms of different DUBs in LUAD to understand the regulatory role of DUBs. In conclusion, the primary function of DUBs in LUAD is to inhibit ubiquitin degradation and stabilize proteins. In addition, DUBs also indirectly affect changes in multiple signaling pathways in the cell. On the other hand, this article primarily discusses whether DUB inhibitors may help treat tumors, especially respect to targeted therapy. Currently, only some small molecule inhibitors are included in the study, and all drug development is still in the preclinical stage. Meanwhile, many DUBs may eventually serve as therapeutic targets. Through these studies, new therapeutic targets have been provided for LUAD targeted therapy, conducive to further drug development.

However, insufficient research on the mechanism of DUBs is a defect of existing studies, resulting in related drug development still being limited. Compared to other types of tumors, such as breast and ovarian cancer, research on DUBs in LUAD is still scarce. Furthermore, substantial work is needed in pharmacology to apply DUBs in clinical therapy. Therefore, in future research on LUAD, DUBs are essential and should be extensively explored.

## Figures and Tables

**Table 1:** The potential functions and mechanisms of DUBs in LUAD (USPs family)

DUBs	Target	Potential functions	Potential mechanisms	Ref
USP4	β-catenin	promote brain metastasis of LUAD cells	stabilize β-catenin protein	74
USP8	PTK	promote the proliferative activity and inhibit apoptosis of tumor cells	the interaction between SFN and USP8 prevents the lysosomal degradation of RTK	49
	EGFR	affect cell proliferation	regulate the expression of EGFR	50
USP9X	PTGES	promote the metastasis of tumor cells	stabilize PTGES	75, 76
USP10	PTEN	inhibit tumor growth and invasion	deubiquitinate and stabilize PTEN	108
	p14ARF	prevent hyper-proliferation and transformation of cells	mediate the deubiquitination of p14ARF	109
USP14	β-catenin	promote proliferation	USP14 contributes to the accumulation of β-catenin protein level	53, 56
USP17	Cyclin A	promote cell cycle from G0 / G1 to S phase	remove the polyubiquitin chains conjugated onto cyclin A and stabilize the cyclin A protein	58
USP18	KRAS	regulating tumor cell proliferation	regulate KRAS protein stability	110
USP22	EGFR	positively regulate cell proliferation and G1 / S phase transformation	stabilize the EGFR protein and increase the recovery and utilization of EGFR	61
USP28	STAT3	promote cell growth	induce STAT3 signaling	111
USP33	PPM1A	suppress EMT	deubiquitinate PPM1A	112
USP37	Snail protein	promote the invasion and migration of LUAD cells	deubiquitinate Snail protein, prevent the degradation of Snail protein	81
USP37	c-Myc	affect the proliferation of cancer cells	deubiquitinate and stabilize c-Myc	82
USP44	PTEN	inhibit cell growth	inhibit AKT signaling by stabilizing PTEN	113
USP49	PTEN	inhibit cell growth	inhibit PI3K/AKT signaling by stabilizing PTEN	114

**Table 2:** The potential functions and mechanisms of DUBs in LUAD (other families)

DUBs	Target	Potential functions	Potential mechanisms	Ref
BAP1		inhibit lung cancer proliferation and promote apoptosis	deubiquitinate host cell factor-1 (HCF1)	65
UCHL1		inhibit cell proliferation	catalyze ubiquityl transfer to a lysine residue (presumably Lys63) on another ubiquitin molecule	115
UCHL3	LSH	influence the invasion and migration of LUAD cells	deubiquitinate LSH	83
OTUB2	U2AF2	promote cell growth, colony formation, migration, and invasive activities	stabilize U2AF2 and activate the AKT/mTOR pathway	116
OTUD3	GRP78	accelerate the growth of cancer cells	deubiquitinate and stabilize GRP78	68
OTUD6B		regulate cell growth and proliferation	OTUD6B-1 represses DNA synthesis while OTUD6B-2 promotes it	117
PSMD14		promote the proliferative activity and inhibit apoptosis of tumor cells	p53-independent p21 down regulation	64

**Table 3:** DUBs inhibitor

Name	Target DUB	Research stage	Ref
ML323 (70)	USP1/UAF1	Preclinical	118
P5091	USP7	Preclinical	119, 120
P22077	USP7	Preclinical	119, 120
WP1130	USP9X	Preclinical	95
Au (PPh3) PT	USP14; UCHL5	Preclinical	105
AgDT	USP14; UCHL5	Preclinical	106
AC17	USP14; UCHL5	Preclinical	107
IU1-47	USP14	Preclinical	56
[1,2,3]triazolo[4,5-d]pyrimidine derivatives	USP28	Preclinical	98
